# An Evaluation of AI‐Generated Clinical Notes in the OpenNotes Era: A Thematic Analysis of Clinician Discourse

**DOI:** 10.1111/jep.70516

**Published:** 2026-06-30

**Authors:** Samuel Atiku, Olufisayo Olakotan

**Affiliations:** ^1^ Research Services Aston University Birmingham UK; ^2^ Digital Technology and Innovation University of Staffordshire UK; ^3^ Department of Neonatology, Women and Children's Directorate University Hospitals Leicester Leicester UK; ^4^ Faculty of Science and Engineering University of Wolverhampton Wolverhampton UK

**Keywords:** artificial intelligence scribes, clinical documentation, netnography, OpenNotes

## Abstract

**Background:**

The integration of ambient artificial intelligence (AI) scribes into the OpenNotes environment presents a profound governance crisis in healthcare. While patient access to medical records was designed as a transparency reform, the introduction of machine‐generated text introduces novel vulnerabilities regarding record integrity, liability, and patients' trust.

**Objective:**

This study investigates how clinicians discursively negotiate the systemic risks and accountability challenges of patient‐facing, AI‐assisted documentation.

**Methods:**

Employing a netnographically informed qualitative design, the research conducted a reflexive thematic analysis of 484 relevant comments across 120 threads from eight clinician‐oriented subreddits spanning October 2020 to February 2026.

**Results:**

The analysis revealed five distinct governance challenges. First, an accountability vacuum exists where the mandatory clinician signature functions merely as a legal shock absorber for institutional AI liability. Second, clinicians frame AI hallucinations as a mathematically inevitable epistemic risk rather than a correctable technical bug. Third, a “dual‐audience” problem emerges, as algorithmic optimization compromises both the individual clinical voice needed for peer communication and the empathetic clarity required for patient readers. Fourth, existing privacy frameworks are structurally inadequate to manage commercial data extraction during patient encounters. Finally, institutional productivity demands and AI‐driven over‐documentation severely threaten the fiscal credibility of the medical record through inadvertent upcoding.

**Conclusions:**

The prevailing regulatory assumption—that a physician's digital signature combined with passive patient visibility guarantees documentation accountability—is a fragile fiction. To protect clinical truth, health systems must transition from models of passive disclosure toward contingent transparency. This requires establishing authoritative, enforceable mechanisms for provenance tracking, error contestation, and vendor accountability.

## 1 Introduction

OpenNotes now occupies the centre of urgent policy debates regarding transparency in clinical care. Patient access to visit notes has fundamentally shifted the medical record away from being an exclusive professional document towards a highly visible part of patient care. A substantial body of research shows that patients value this access because it improves understanding, supports recall, strengthens engagement, and fosters a greater sense of partnership with clinicians [1, 2, 3, 4]. Conversely, note access can generate confusion, distress, privacy concerns, and disputes over perceived errors, especially where patients fear being seen as difficult [5, 6, 7]. Consequently, OpenNotes represents a profound governance reform because it dictates who sees documentation and how healthcare organisations must respond.

These questions are increasingly acute as patient access becomes firmly embedded in federal regulation. The information blocking framework associated with the 21st Century Cures Act in the United States has strengthened expectations for free data access, while concurrent federal guidance has clarified strict enforcement mechanisms for actors who improperly interfere [8, 9]. Patient portals are no longer discretionary local innovations. They are the mandated operating logic of modern digital care.

A significant complication has rapidly entered this regulatory landscape. AI scribes are being deployed aggressively to address the well‐documented burden of electronic health record documentation. Prior research clearly demonstrates that documentation work is associated with profound workflow disruption, cognitive burden, burnout, and the displacement of clinically valuable tasks [10, 11, 12]. Early reviews and a subsequent randomised trial of these AI tools suggest they reduce writing time but simultaneously raise severe concerns regarding note quality, editing burden, systemic integration, and the absolute need for human oversight [13, 14, 15]. Once machine‐generated notes are shared directly with patients, technical limitations immediately escalate into patient‐facing transparency crises.

This paradigm shift rewrites the transparency debate. Earlier research suggested that patient access alters documentation style, clinician candour, and fundamental perceptions of the medical record [6]. More recent work in mental health settings similarly reports reduced jargon, more explanatory detail, and perceived added workload following the implementation of open notes [16]. Artificial intelligence intensifies these exact tensions by introducing profound uncertainty about data provenance and clinical accuracy precisely when documentation is most open to public scrutiny.

The contingent payoff framework offers a vital policy lens for interpreting this structural problem. Atiku argues that passive transparency rarely produces systemic change on its own, noting that stronger effects only occur where transparency is intimately linked to meaningful opportunities for challenge or credible enforcement mechanisms [17]. Patients frequently read notes and identify mistakes while entirely lacking practical routes to correction. Bell et al. (2020) found that patients routinely identify clinically serious errors in ambulatory notes, while Lam et al. (2021) showed that institutional barriers to speaking up remain pervasive [7, 18]. The prevailing system remains passive, where arrangements for correction are legally weak. Machine‐generated text heavily compounds this governance failure.

The convergence of OpenNotes and artificial intelligence must be treated as a critical site of policy intervention. Digital efficiency claims lose credibility once rigorous proofreading, dispute handling, defensive documentation, and patient confusion are accurately measured as institutional governance costs [19]. This study examines online professional forums where these issues are actively negotiated. The research asks how clinicians discursively manage the governance challenges arising when patients access machine‐generated notes. Furthermore, it explores what these negotiations reveal about accountability, care quality, and fiscal credibility.

Existing rules for medical accountability remain structurally inadequate. Current policy arrangements rely on a deeply flawed assumption that a clinician's signature, combined with passive patient access, is sufficient to protect record integrity. Unchecked hallucinated content and algorithmic documentation shaped primarily by billing pressures can now easily enter the legal record. Health systems and regulators must urgently establish stronger standards for data provenance, human review, patient correction, and vendor accountability to achieve true clinical transparency.

## 2 Methodology

### 2.1 Research Design

This study adopted a netnographically informed qualitative design combining computational retrieval of Reddit discussions with reflexive thematic analysis. Netnography was appropriate because the research question concerned how governance challenges around OpenNotes, AI‐generated clinical notes, accountability, privacy, and documentation burden are discursively negotiated in online professional spaces. Rather than treating Reddit as a store of opinions, the study approached it as an interactional environment in which healthcare workers and other healthcare‐adjacent participants publicly interpret, contest, and stabilise meanings around note‐writing, patient visibility, medico‐legal risk, and AI adoption. In this sense, the study drew on netnography's concern with naturally occurring online discourse and shared meaning‐making [20].

The analysis was also informed by reflexive thematic analysis because the aim was interpretive rather than enumerative. The purpose was not to count attitudes toward AI scribes or OpenNotes, but to identify patterned ways participants constructed accountability, epistemic risk, note quality, privacy, and fiscal credibility across discussions. Reflexive thematic analysis was well suited to this aim because it supports conceptually rich theme development while recognising the active role of the researcher in coding and interpretation [21, 22].

### 2.2 Data Source and Sampling Frame

Reddit was selected because it provides access to public, pseudonymous, interactional discourse that is often less filtered than interview or organisational material. This mattered because clinicians and healthcare workers may discuss documentation burden, institutional frustration, privacy concerns, and medico‐legal anxieties more candidly in online forums than in formal settings. Online discussion forums are methodologically valuable because they preserve disagreement, uptake, and co‐construction in ways that are analytically useful for discursive research [23].

The initial sampling frame comprised 13 clinician‐oriented and healthcare‐adjacent subreddits: r/medicine, r/Residency, r/nursing, r/medicalschool, r/emergencymedicine, r/healthIT, r/hospitalist, r/FamilyMedicine, r/IntensiveCare, r/Noctor, r/psychiatry, r/AskDocs, and r/healthcare. These were chosen to maximise variation across specialty, role, training stage, and documentation setting. The aim was not statistical representativeness, but analytic range and information richness, consistent with qualitative sampling principles and the logic of information power [24].

The search window ran from 1 January 2016 to February 2026. This broad frame was intended to capture discussion spanning the implementation of patient‐facing note access and the later uptake of ambient AI scribing tools. In practice, the earliest substantively relevant retained thread dates from 2020. Although all 13 subreddits were queried, the final retained corpus came from eight communities: r/FamilyMedicine (36 threads), r/medicine (27 threads), r/healthIT (22 threads), r/Residency (15 threads), r/emergencymedicine (14 threads), r/medicalschool (3 threads), r/hospitalist (2 threads), and r/nursing (1 thread).

### 2.3 Search Strategy and Data Extraction

Data were collected using a custom Python script built with the Python Reddit API Wrapper (PRAW). Programmatic extraction was necessary because the study required systematic searching across multiple subreddits, reproducible query logic, preservation of thread hierarchy, and export of structured metadata for later qualitative analysis.

The search strategy was organised around three overlapping domains: OpenNotes and patient access, AI‐assisted or ambient documentation, and chart correction or patient challenge. To improve recall, the script used both grouped keyword families and five Boolean/Lucene‐style search strings. These included terms such as “open notes,” “opennotes,” “patient portal,” “ai scribe,” “ambient ai,” “generated note,” “dragon copilot,” “abridge,” “nabla,” “correct the note,” “amend the chart,” and “asked me to change the note.” This multi‐query design was deliberate, since clinicians do not always use formal policy language such as “OpenNotes,” and relevant discussions often appear through adjacent phrases like “patient read my note” or “patient portal.”

For each subreddit, the script first searched these terms using Reddit search, sorted by new, with a limit of 120 posts per query and all‐time filtering. If this did not yield enough candidate material, the script then searched subreddit new posts and top posts. To prevent domination by a small number of large communities, the corpus was capped at 120 threads, with a maximum of 40 threads per subreddit. This was a pragmatic balancing decision intended to preserve diversity while keeping the corpus manageable for deep qualitative analysis. In line with reflexive thematic analysis, adequacy was justified by thematic richness and information power, not by statistical representativeness or simplistic claims of saturation [22, 24].

For each qualifying submission, the script extracted the entire comment tree, preserving chronological order, parent‐child structure, depth, and comment path. This was methodologically important because the thread, rather than the isolated comment, was the primary unit of analysis. Governance claims were often produced dialogically, through challenge, rebuttal, elaboration, and practical settlement across comment chains [23].

### 2.4 Inclusion, Exclusion, and Analysis

Screening proceeded in two stages: automated filtering followed by manual validation. The script excluded comments under 30 characters, AutoModerator content, duplicate thread retrievals, and NSFW posts. It also generated a boolean relevant_to_topic flag based on terms associated with OpenNotes, AI‐generated notes, correction, hallucination, proofreading, or defensive documentation. Across the 120 retained threads, the script collected 3577 comments in total, of which 516 were flagged as potentially relevant.

Manual review then determined the final analytic corpus, because keyword matching alone could not reliably distinguish substantively relevant material from incidental mention. The final manually validated corpus comprised 120 threads and 484 analytically relevant comments.

The data were analysed using thread‐level reflexive thematic analysis focused on how participants assigned responsibility, narrated hallucination and misattribution, discussed patient visibility, described privacy and consent concerns, and linked technical problems to wider legal, organisational, and economic pressures. Reddit scores were retained only as contextual metadata, not as measures of truth, prevalence, or representativeness.

### 2.5 Ethical Considerations

The study used publicly accessible Reddit content and involved no interaction with users. However, public accessibility does not remove ethical responsibility. Current internet research ethics guidance recommends a context‐sensitive approach to online data, especially where pseudonymous content remains searchable and potentially identifiable [25]. Accordingly, the study focused on discourse patterns rather than personal biography and treated quoted material cautiously in light of traceability risks.

## 3 Results

This results section presents findings from a thematic analysis of 484 relevant comments drawn from 120 Reddit threads posted between October 2020 and February 2026. The dataset spans key clinical subreddits including r/FamilyMedicine, r/medicine, r/healthIT, r/Residency, r/emergencymedicine, r/medicalschool, r/hospitalist, and r/nursing. The corpus captures the discursive arc from the implementation of US federal information‐blocking requirements supporting patient access to clinical notes through the rapid proliferation of ambient AI scribing technologies. Rather than treating individual posts as isolated data points, we traced argumentative trajectories across linked comment chains. We attended to how threads collectively constructed, contested, and stabilised governance positions through cumulative multi‐voice discourse.

Five overarching themes emerged. Each is presented as a narrative analytical section, grounding the theme in thread‐level dynamics. This is followed by a structured evidence table presenting twelve supporting quotations with source details and analytical notes. Quotes are identified by commenter handle, thread title, and Reddit score. The score serves as a proxy for community endorsement within the discourse.

### 3.1 Theme 1. the Accountability Vacuum: Clinicians as Final Arbiters of AI‐Authored Text in the Legal Medical Record

The most pervasive governance concern across the corpus is accountability when artificially generated text enters the legal record (Table 1). Threads collectively construct a discursive settlement positioning the signature as the definitive governance anchor. Users note that “as soon as you hit sign, that hallucination becomes your responsibility” (Q1), marking the exact transfer of institutional liability to the individual practitioner. However, this settlement fractures under practical scrutiny because “proofreading is disincentivized in any model which rewards productivity” (Q2), converting individual accountability into an unrealisable demand.

**Table 1:** Illustrative quotations for Theme 1: accountability, clinician oversight, and liability in AI‐generated clinical notes.

#	Quote	Source & score	Analytical note
1	“You ALWAYS proofread. If you get complacent, you'll miss something, and as soon as you hit sign, that hallucination becomes your responsibility.”	Commentator 1 T5: Which AI scribe gets it right? Score: 51	Establishes the signature as the governance anchor — the legal transfer point at which AI liability shifts entirely to the individual clinician. Foundational to the accountability settlement.
2	“AI notes are untrustworthy garbage and I never know if the things that they're saying are actual reality, or just the AI's conception of how things should be or typically are. Proofreading is disincentivized in any model that rewards productivity above everything else.”	Commentator 2 T34: AI Scribes Score: 27	Fractures the settlement by naming its structural failure condition: productivity incentives make a thorough review economically irrational, converting individual accountability into an unrealisable governance demand.
3	“I've had the AI scribe say that I said there was xyz% chance of response/cure, but when I reviewed the recording/transcript, it was the patient speculating/asking a question — not anything that I said.”	Commentator 3 T10: I, for one, welcome our robot overlords Score: 86	Introduces misattribution of speaker as a distinct accountability class: the AI places the patient's words in the clinician's mouth, creating fabricated evidence of a clinical commitment never made.
4	“This is exactly the error—misattribution of who said something in a meeting with a patient—that was reported by a therapist over on r/therapist using an AI scribe, that she belatedly found out was rife in her charts.”	Commentator 4 T10: I, for one, welcome our robot overlords Score: 3	Cross‐subreddit citation extending accountability discourse into therapy. “Belatedly found out was rife” describes systematic error discovered after‐the‐fact, during which AI misattributions constituted the legal record.
5	“Just had TOC with wife/husband—she was admitted for an acute ortho issue, he had a GI bleed recently. So note looked like she had ortho issue and GI bleed — it all sounded reasonable when Abridge reported it, but was so far from what happened I was stunned.”	Commentator 5 T5: Which AI scribe gets it right? Score: 1	“It all sounded reasonable” is the central epistemological problem: plausible‐sounding but factually incorrect documentation. The note did not flag its own error; only the clinician's direct knowledge revealed the fabrication.
6	“The problem is these AI scribe tools are black boxes and you can't learn WHY some wildly bad notes are so off.”	Commentator 6 T5: Which AI scribe gets it right? Score: 1	Names the epistemological barrier that prevents learning from error. When the source of failure cannot be traced, the professional feedback loop that sustains accountability breaks down entirely.
7	“As a patient: I have an accent and AI misinterpreted what I said half the time, and no one bothered to correct it. I was hospitalized for disseminated zoster, but the AI wrote a whole thing about HIV and possible mechanisms of transmission.”	Commentator 7 T104: Do AI scribes help with documentation time? Score: 9	Patient testimony functioning as accountability disruption: the governance framework (proofreading prevents harm) collides with documented real‐world harm. An accent error compounded through AI reasoning into clinically significant misinformation.
8	“All too often, I still see glaringly obvious mistakes with dictation and AI scribes.”	Commentator 8 T94: Anyone not using dictation or AI scribe? Score: 23	“Glaringly obvious” implies errors that survived proofreading. This counter‐voice, appearing in a thread of AI adopters, suggests that the review‐as‐safeguard model fails even when review is nominally performed.
9	“This note has two different “history of presenting illness” sections. The poor shmuck stuck reading it has lost 5 min of their life.”	Commentator 9 T27: AI generated A&P are hot garbage Score: 32	“The poor shmuck stuck reading it” extends accountability beyond the signing clinician to downstream readers—consulting physicians and covering staff who bear cognitive burden from inflated AI prose.
10	“The majority of my A& Ps I leave as AI writes it.”	Commentator 10 T12: AI Scribe Score: 5	Read against the accountability settlement, this represents the governance failure point: when review is “occasional” rather than systematic, accountability becomes aspirational rather than operational.
11	“If you are willing to accept what might be some substandard documentation, the AI‐generated notes save a lot of time. If you are particular about what winds up in your note, you will probably spend about as much time reviewing and correcting as you would have if you had just done it yourself.”	Commentator 11 T104: Do AI scribes help with documentation time? Score: 23	The accountability trade‐off is made explicit: time savings require accepting substandard documentation. “If you are willing to accept,” converts a governance question into a personal preference, individualising a structural failure.
12	“As a person who is developing AI tools for medical things… I've seen it hallucinate so much that I feel like at this point I myself may be an AI hallucination.”	Commentator 12 T104: Do AI scribes help with documentation time? Score: 6	A developer's sardonic self‐erasure as critique: when even builders cannot reliably distinguish fabrication from reality, the oversight burden placed on individual clinicians during note review is structurally unrealistic.

The discourse revealed distinct accountability crises, most notably insidious speaker misattribution. Algorithms fabricate clinical commitments by putting patient speculation directly into the clinician's mouth (Q3), an error discovered belatedly and systematically in therapy records (Q4). The central epistemological challenge is that these fabrications are functionally invisible because they “all sounded reasonable” (Q5), making them indistinguishable from accurate documentation without immediate access to the original encounter. Because these opaque “black box” tools (Q6) prevent professionals from learning why notes fail, theoretical safeguards frequently collide with documented patient harm, such as uncorrected accent misinterpretations compounding into clinically significant misinformation (Q7).

Consequently, when “glaringly obvious mistakes” (Q8) survive nominal review, the cognitive burden of inflated prose transfers to downstream consulting colleagues (Q9). When time pressures force clinicians to accept “substandard documentation” (Q11) or leave notes “as AI writes it” (Q10), the oversight mandate becomes structurally unrealistic (Q12). In summary, legal frameworks insist on individual liability, yet practice realities render human oversight impossible, ultimately reducing the clinician to a mere legal shock absorber for institutional technological failures.

### 3.2 Theme 2. Hallucination as Governance Crisis: Epistemic Risk, Mathematical Inevitability, and the Limits of Individual Review

Hallucination emerged across the corpus as the central epistemic governance challenge distinguishing algorithmic documentation from prior clinical records (Table 2). Rather than treating these errors as correctable software bugs, clinicians frame hallucinations as a mathematical inevitability based on training processes (Q1). Industry developers confirm this view by characterising the phenomenon as an inherent and unsolvable issue requiring managed acceptance (Q2). Consequently, the discourse reveals an undeniable and unfixable risk (Q3) that forces healthcare systems to transition from pursuing perfect accuracy toward permanent risk management.

**Table 2:** Illustrative quotations for Theme 2: Hallucination, epistemic risk, and the limits of individual review.

#	Quote	Source & score	Analytical note
1	“OpenAI recently released a study stating that large language model hallucination is a mathematical inevitability based on the training process. Guessing is rewarded instead of uncertainty and thus hallucinations will occur.”	Commentator 13 T35: Open Evidence examples of AI hallucination? Score: 73	Establishes hallucination as a mathematical property of LLM architecture, not a correctable quality‐control failure. Shifts governance from “AI improvement” to “permanent non‐zero error rate management.”
2	“ ‘Hallucinations’ are an inherent and non‐solvable issue with Large Language Models—I say this as someone who's architected and developed an ambient scribe solution. Being “cut off from the outside” will not lessen hallucinations.”	Commentator 14 T82: Patient question—how secure is the Epic AI module? Score: 5	Developer insider confirming non‐solvability. “Lean in to the limitations” proposes managed acceptance as a governance posture: educational and institutional responses must be structured around known permanent error risk.
3	“If it's based on an LLM, then there is inherent, undeniable, and unfixable risk of hallucination—I just wonder how pervasive it is.”	Commentator 15 T10: I, for one, welcome our robot overlords Score: 113	Score 113 makes this among the corpus's most endorsed comments. “Undeniable and unfixable” forecloses technical solution, forcing a governance response that must operate independently of AI improvement.
4	“I have seen inaccurate application of citations—suggesting a treatment for one condition but citing a paper about a different condition or patient population. I have stopped using it altogether.”	Commentator 16 T35: Open Evidence examples of AI hallucination? Score: 76	Distinguishes active hallucination (fabrication) from passive omission (excluding relevant studies)—two failure classes requiring different oversight strategies. Exit rather than mitigation as the individual governance response.
5	“DAX does a HORRIBLE job for anything remotely complex: missing important details in the HPI and even whole problems sometimes in the plan. It sometimes hallucinates physical findings.”	Commentator 17 T7: Experience With AI Scribe Thus Far Score: 5	Hallucinated physical findings constitute fabrication of clinical evidence—qualitatively distinct from stylistic errors. Maps the hallucination risk gradient from low‐risk simple visits to high‐risk complex presentations.
6	“Today's gem: “Patient name is a 77‐year‐old male who presents for follow‐up regarding his new medication, Kyleena.” (Actual new med—Kerendia.)	Commentator 18 T27: AI generated A&P are hot garbage Score: 4	Kyleena (hormonal IUD) vs Kerendia (mineralocorticoid antagonist for CKD)—entirely different drug classes and patient populations. Phonological plausibility masking clinical nonsense illustrates why fluent AI text cannot be efficiently reviewed without encountering knowledge.
7	“It's really not that great for psych. Both AI scribes I've tried have had a terrible time identifying actual relevant information. “Saying a lot without actually saying anything” is how I describe it.”	Commentator 19 T10: I, for one, welcome our robot overlords Score: 15	Psychiatric HPIs require nuanced symptom capture supporting therapeutic continuity and risk management. The failure to distinguish clinically salient from incidental speech is particularly dangerous in mental health documentation.
8	“Saw a kid with a metabolic disease—it added every single manifestation of the disease as a separate problem with its own plan of diet and meds, while capturing none of our half‐hour discussion of the disease and its natural history.”	Commentator 20 T7: Experience With AI Scribe Thus Far Score: 41	AI pattern‐matching lists all known disease manifestations rather than documenting the specific discussion. The note over‐claims by listing problems not discussed while under‐capturing actual clinical reasoning communicated to the family.
9	“The more I know about a subject, the more there are small inconsistencies or errors in AI's answers. What is invariably true is that the AI's answer will sound good—which is the function LLMs excel at.”	Commentator 21 T35: Open Evidence examples of AI hallucination? Score: 3	Error detection is expertise‐dependent. The more specialised the domain, the higher the detection burden. Governance frameworks relying on individual review have systematically higher failure rates in specialist settings.
10	“Hallucinations or errors are fairly common, though—you always need to review the transcripts.”	Commentator 22 T10: I, for one, welcome our robot overlords Score: 37	“Fairly common” juxtaposed with “always review” creates a governance bind: if errors are common, review must be thorough enough to catch them, which undermines the time‐saving rationale for AI adoption.
11	“I've never encountered or heard of a hallucination. It will occasionally make mistakes where it attributes something the patient said to something I said, or replaces one medical term with a similar‐sounding but different term—but I attribute this mostly to how patients pronounce things.”	Commentator 23 T10: I, for one, welcome our robot overlords Score: 10	Redefines hallucination narrowly, reclassifying misattribution and phonological substitution as “occasional mistakes.” This definitional narrowing makes low hallucination rate claims depend on what one counts as a hallucination.
12	“I never make my corrections to the AI scribe just so AI stays a little crazy and we stay a little ahead. I have found so many errors in OpenEvidence that I don't trust it for medical decision making.”	Commentator 24 T6: Are we training our replacements? Score: 1	Deliberate non‐correction of AI errors as a competitive advantage preservation. Individually rational but collectively a governance failure — allowing error‐prone AI to remain in clinical use rather than improving it through feedback.

Threads construct a concrete failure typology differentiating passive omissions from active hallucinations, such as the inaccurate application of citations (Q4) or the outright fabrication of physical findings during complex encounters (Q5). Phonological plausibility frequently masks clinical nonsense. Algorithms will confidently substitute entirely different drug classes, creating fluent but dangerous text that cannot be efficiently reviewed without direct encounter knowledge (Q6). This epistemic risk is highly contextual. In psychiatric intake, algorithms fail to identify actual relevant information (Q7), while in paediatric care, pattern recognition captures generic disease manifestations rather than the specific clinical discussion (Q8). Because the artificial output will invariably sound good, error detection becomes heavily dependent on expertise (Q9).

This dynamic creates a profound governance bind. If errors are fairly common, clinicians must invariably review transcripts (Q10), which entirely undermines the intended administrative relief. Some users respond through definitional narrowing of what constitutes an error (Q11) or adopt perverse strategies of deliberate noncorrection (Q12). Ultimately, algorithms that confidently generate functionally invisible errors severely destabilise care quality under the OpenNotes mandate, exposing patients to direct epistemic harm.

### 3.3 Theme 3. Note Quality, Clinical Voice, and Documentation as Professional Practice: The Dual‐Audience Problem of AI Notes in an OpenNotes Era

The convergence of AI scribing with the OpenNotes mandate fractures the communicative purpose of the medical record (Table 3). Clinicians argue that note quality extends beyond factual accuracy to encompass clinical reasoning, transparency, and patient accessibility. A strong thread‐wide consensus reveals an asymmetry in AI output: while it succeeds on narrative history, the assessment and plan sections are often “borderline useless” (Q2). This is a critical governance finding, as these sections are directly implicated in care continuity and medicolegal protection.

**Table 3:** Supporting quotations for Theme 3: Note quality and clinical voice.

#	Quote	Source & score	Analytical note
1	“I was so excited to start using our new built‐in AI scribe—until I read some of my partners' notes. I'll just keep doing Dragon.”	Commentator 25 T27: AI generated A&P are hot garbage Score: 168	Score 168—among the corpus's most endorsed. Promise‐to‐disappointment arc driven by reading peers' output. Introduces the colleague‐reader as a quality governance actor, not just the signing clinician.
2	“A&P and physical are borderline useless with Abridge at least.”	Commentator 26 T27: AI generated A&P are hot garbage Score: 54	Thread‐wide consensus position: AI succeeds in narrative history but fails in clinical reasoning documentation. Critical because A&P is most directly implicated in care continuity, billing justification, and medicolegal protection.
3	“90% chance their notes are now completely bloated trash since they don't proofread jack shit.”	Commentator 27 T27: AI generated A&P are hot garbage Score: 22	Community‐level quality audit distributing AI adopters into two populations: technically sophisticated optimisers and uncritical producers of “bloated trash.” Names the governance problem of uneven adoption standards across the same institution.
4	“I went back to read my notes, and they sound nothing like me. Nothing is wrong, except they're a little wordy and the narrative flow is weird. They just sound like AI. I felt nervous that I missed something the AI scribe wrote erroneously.”	Commentator 28 T107: Seeing 30 patients a day with AI scribes? Score: 34	“Sound nothing like me” names the loss of clinical voice—the individualised prose through which experienced clinicians communicate judgement and nuance. Generic AI prose removes the signal that allows colleague‐readers to calibrate interpretive weight.
5	“I feel like having an AI listen and take my notes for me will make me check out on consolidating what the patient actually says down into relevant information for the chart, leading to note bloat. Additionally, I think there will be legal ramifications in medmal cases, regarding how the chart looks.”	Commentator 29 T10: I, for one, welcome our robot overlords Score: 19	Links note quality to the cognitive process of documentation: writing forces selection and synthesis that AI ambient recording bypasses. The medicolegal concern connects quality governance directly to liability governance.
6	“I can often remember a patient even a significant time later by reading my note—AI scribes aren't naturally going to include a unique identifying characteristic and are naturally going to make notes that look similar.”	Commentator 30 T7: Experience With AI Scribe Thus Far Score: 1	Notes as memory prosthetics, enabling clinicians to reconstruct the particularity of past encounters. AI‐generated notes that look “similar” to each other fail this function, impairing care continuity for follow‐up visits.
7	“Patients find it offensive when what is documented does not match their recollection of the visit. They may also react to words like obesity and commonly used phrases like “patient denies” or “patient complains.”	Commentator 31 T2: AMA—OpenNotes clinicians and researchers Score: 3	Establishes the quality baseline for patient‐readable documentation. AI tools that default to clinical vocabulary without patient‐audience awareness produce technically accurate but discursively alienating notes, failing the dual‐audience requirement.
8	“In some ways, it might even improve care a little—I can't just speculate about the effects of a patient's obesity behind their back, I have to address it in the exam room knowing there's a slight chance they might read my note.”	Commentator 32 T1: Open Notes—Open Bills need to happen too Score: 13	OpenNotes as a quality governance mechanism: patient readability creates accountability for clinical content. For AI notes, the question is whether AI scribing tools can reproduce this self‐regulatory awareness without explicit instruction.
9	“Oh man, open notes for ED visits would be utter chaos, doubly so if they come with some easy way for the patient to communicate dissatisfaction.”	Commentator 33 T3: Thoughts on Open Notes legislation Score: 18	Emergency documentation contains time‐pressured, frank assessments serving different communicative functions than outpatient notes. AI‐generated ED notes inherit this tension while making frank assessments more legible—and alarming—to patient readers.
10	“Eventually, you'll just use AI to parse other people's verbose AI‐generated notes.”	Commentator 34 T13: I tried a lot of AI medical scribes Score: 9	AI‐to‐parse‐AI as system‐level quality critique. If AI scribing becomes the default, the documentation environment degrades for every colleague. Individual adoption imposes a quality externality on the entire healthcare team.
11	“There should be AI‐generated notes to appease our billing overlords. And then a section where physicians can just free‐text pertinent information for other physicians.”	Commentator 35 T27: AI generated A&P are hot garbage Score: 5	Two‐tier note proposal: AI layer for compliance, human layer for clinical communication. Directly addresses the dual‐audience problem by proposing structural separation rather than attempting both purposes with one instrument.
12	“You can be honest without being condescending.”	Commentator 36 T1: Open Notes—Open Bills need to happen too Score: 132	Encapsulates the quality standard for dual‐audience documentation in six words. For AI scribing tools, the governance question this raises is whether they can be instructed to apply this standard across clinical vocabulary and patient readability simultaneously.

The promise of AI frequently collides with the reality of reviewing colleagues' output, leading clinicians to revert to prior practices after reading “bloated trash” produced by uncritical peers (Q1, Q3). Furthermore, clinicians report profound alienation from their own records, noting that AI‐generated prose “sounds nothing like me” (Q4). This loss of individualised clinical voice removes the crucial signal that allows colleagues to calibrate interpretive weight. Because AI standardisation creates notes that “look similar” (Q6), they fail to function as individual memory prosthetics. Moreover, AI ambient recording bypasses the cognitive selection and synthesis process that inherently constitutes clinical reasoning (Q5).

Patient accessibility introduces a new quality governance dimension. Tools trained on clinical corpora often fail the “dual‐audience requirement” by producing alienating phrasing that patients find “offensive” (Q7). Clinicians thus face a severe communicative bind, seeking notes that are “honest without being condescending” (Q12). Rather than resolving this, the discourse suggests a system‐level degradation where clinicians eventually “use AI to parse other people's verbose AI‐generated notes” (Q10). Ultimately, the clinical voice is sacrificed to algorithmic throughput, prioritising institutional metrics over therapeutic relational value.

### 3.4 Theme 4. Privacy, Data Sovereignty, and the HIPAA Governance Gap: Commercial AI Tools, Consent Practices, and the Structural Inadequacy of Existing Frameworks

Commercial artificial intelligence scribes expose a severe regulatory failure regarding patient data sovereignty (Table 4). Frameworks like HIPAA are structurally inadequate when data extraction operates as a primary business model. Clinicians view algorithmic training as uncompensated value extraction, noting they are often “training AI for free” (Q1). Traditional deterrence fails because venture‐backed startups “consider fines and lawsuits part of their current business expenses” (Q2). Consequently, practitioners establish the Business Associate Agreement as a binary threshold, warning peers to “DO NOT use one” without it (Q3).

**Table 4:** Supporting quotations for Theme 4: Privacy, consent, and data governance.

#	Quote	Source & score	Analytical note
1	“I have liability concerns—both HIPAA and medicolegal. You're training AI for free (or even paying to use it). You are training your replacement.”	Commentator 38 T4: What AI scribe are you using and why? Score: 28	Data training as value extraction: conversion of de‐identified clinical encounter data into proprietary model improvements. HIPAA frameworks do not address this structural privacy concern; compliance certificates do not resolve it.
2	“I trust EMRs about as far as I can throw them, but at least they're afraid of the HIPAA hammer too. These AI guys consider fines and lawsuits part of their current business expenses because there's no government regulations holding them back with real threats.”	Commentator 39 T4: What AI scribe are you using and why? Score: 7	HIPAA as coercion rather than culture. Deterrence does not function the same way for venture‐backed startups that price regulatory risk into business models as for established healthcare institutions with reputational stakes in compliance.
3	“Unless you're using a software promoted by your own hospital or you are in private practice, able to arrange a HIPAA BAA prior to using an AI scribe programme, DO NOT use one.”	Commentator 40 T40: Has anyone tried AI scribes? Score: 1	The BAA as binary governance criterion: its presence permits use; its absence prohibits it. All‐caps “DO NOT” performs community governance norm while revealing absence of institutional enforcement—clinicians policing peer behaviour.
4	“When we use these scribes, we often trade long‐term data sovereignty for short‐term administrative relief. If a company isn't explicitly transparent about their “Zero‐Retention” policy, assume the worst.”	Commentator 41 T6: Are we training our replacements? Score: 1	“Assume the worst” inverts the default from trust‐until‐violated to scepticism‐until‐proven, reflecting epistemic limitations clinicians face when evaluating opaque vendor claims. Position privacy governance as a professional concern, not just patient protection.
5	“Their policy lets de‐identified or aggregated patient data be used to “improve the platform.” Data can be shared with third‐party or overseas providers. Patient data might be handled in ways your patients haven't consented to.”	Commentator 42 T34: AI Scribes Score: 1	Identifies specific mechanisms—de‐identification for training, third‐party sharing, overseas processing—that individually comply with HIPAA while collectively enabling re‐identification and data repurposing without patient consent.
6	“From my perspective, according to Fourth Amendment property rights, they shouldn't be able to use your data to do this, but they do.”	Commentator 43 T6: Are we training our replacements? Score: 1	Constitutional governance framework beyond HIPAA's regulatory structure. The resigned “but they do” acknowledges that legal norms and commercial practices are misaligned and that existing enforcement cannot close the gap.
7	“Locally, we can't use an AI scribe without your consent. This isn't true everywhere at the moment, but likely will be in the future. Most companies do not keep a transcript of the recording.”	Commentator 44 T13: I tried a lot of AI medical scribes Score: 3	Maps geographic and institutional variation in consent requirements. Anticipatory governance posture: stronger consent frameworks treated as an emerging norm rather than a current requirement, predicting regulatory convergence.
8	“Are your patients aware that you're using Abridge? Do they need to agree before you can use it?”	Commentator 45 T110: Piloting Abridge, the AI scribe Score: 1	A basic patient consent question that goes unanswered in Thread 110—a silence that is itself a governance finding. Marks the outer boundary of the thread's governance settlement: community norms around patient notification have not stabilised.
9	“Data privacy in AI scribes is a real concern, especially for therapy sessions. Tools like Freed let you generate notes without feeding your sessions back into their training models.”	Commentator 46 T6: Are we training our replacements? Score: 2	Therapy sessions present the highest‐sensitivity privacy scenario. Positions “no training” as a premium privacy feature, implying that the default assumption—AI training on session data—is the governance baseline requiring explicit exception.
10	“DAX Copilot is fully HIPAA compliant. Add in the security from MSFT's platform and it's a pretty airtight system.”	Commentator 47 T82: Patient question—how secure is Epic's AI module? Score: 15	HIPAA compliance plus enterprise cloud security offered as a compound privacy certificate. Institutional backing (Microsoft, Epic) carried more discursive weight than vendor self‐certification—yet subsequent comments continued to raise accuracy and consent concerns.
11	“ ‘The cloud’ is often used as vague shorthand when patients deserve specifics. You have every right to ask detailed questions about where your health data goes.”	Commentator 48 T82: Patient question—how secure is Epic's AI module? Score: 3	Critique of “the cloud” as vague shorthand names the information asymmetry, making meaningful patient consent impossible without specific disclosure. Implies institutional obligation to standardise what clinicians tell patients about AI data handling.
12	“I don't think we, speaking as patients, can totally stop our physicians from using AI, in as much as no teacher nowadays can stop pupils from using AI to some degree.”	Commentator 49 T13: I tried a lot of AI medical scribes Score: 2	Dual clinician‐patient positionality reconfigures privacy governance as a personal stake. The normalisation analogy implies consent regimes must be designed around the assumption of AI use rather than the possibility of patient opt‐out.

Faced with opaque policies, clinicians advocate profound caution. They advise colleagues to “assume the worst” regarding retention (Q4). This scepticism is justified by policies allowing deidentified data to reach overseas providers, meaning information is “handled in ways your patients haven't consented to” (Q5). Practitioners acknowledge a stark misalignment with fundamental property rights, noting vendors “shouldn't be able to use your data to do this, but they do” (Q6).

Consent requirements currently vary by geography (Q7). Basic questions about whether patients “need to agree before you can use it” routinely go unanswered in professional spaces (Q8). In sensitive contexts like therapy, generating records “without feeding your sessions back into their training models” is treated as a premium feature (Q9). While some rely on overarching institutional compliance certificates (Q10), others critique vague terms like “the cloud” because patients deserve specific details about data destinations (Q11). Ultimately, stakeholders recognise the structural inevitability of these algorithms, comparing them to how “no teacher nowadays can stop pupils from using AI” (Q12). Legal compliance no longer equates to ethical data stewardship.

### 3.5 Theme 5. Fiscal Credibility and the Economic Governance of AI Documentation: Throughput, Coding Accuracy, and the Structural Economics of Clinical Productivity

The uptake of AI scribes in clinical practice is shaped not only by hopes of administrative relief, but also by the economic and coding requirements embedded in contemporary documentation systems (Table 5). Across the corpus, clinicians frequently decomposed the value proposition of these tools, and several questioned whether they meaningfully increased patient throughput. Participants noted that AI “didn't make it any easier to see more patients” (Q1) and did “not increase the number of patients I review per day” (Q2). Rather than framing AI scribes primarily as engines of speed, many described their value in relation to documentation completeness, coding support, and the capture of language needed to satisfy payment and compliance requirements, including hierarchical condition category (HCC) coding (Q3). In this sense, the discourse suggests that AI adoption is tied as much to the demands of billing systems as to clinical workflow itself.

**Table 5:** Supporting quotations for Theme 5: Fiscal credibility and billing integrity.

#	Quote	Source & score	Analytical note
1	“Honestly, AI scribes made my notes better and I think they made me a better doctor, but didn't make it any easier to see more patients.”	Commentator 50 T107: Seeing 30 patients a day with AI scribes? Score: 83	Directly addresses the throughput promise and finds it partially false. Decomposition—better notes, better doctor, not more patients—separates documentation quality benefits from productivity benefits, challenging the primary fiscal rationale for institutional investment.
2	“AI scribes: improvement documentation. Make me feel less tired/fed up at the end of the day. Do not increase the number of patients I review per day. Do not make me a better doctor.”	Commentator 51 T107: Seeing 30 patients a day with AI scribes? Score: 51	Enumerated self‐assessment validates exactly one of four AI scribing claims: documentation quality improvement. Explicitly rejecting physician quality improvement alongside throughput improvement challenges two of the three pillars of the AI scribing value proposition.
3	“The notes are cleaner and more complete with AI scribes, but it hasn't really turned into a “30 patients/day machine.” Where I've noticed a real difference is in HCC coding, making sure guideline language is in there.”	Commentator 52 T107: Seeing 30 patients a day with AI scribes? Score: 28	HCC coding benefits as a revenue rationale independent of visit volume. AI scribes that reliably capture guideline language may have substantial risk‐adjustment payment implications—a fiscal governance claim that bypassed the quality debate.
4	“AI scribes don't save me noticeable time personally. Note, I am in Canada, so simple patients are only getting a sentence or two of documentation. Not having to write essays just to meet billing requirements.”	Commentator 53 T10: I, for one, welcome our robot overlords Score: 50	Canadian comparison externalises the US billing documentation problem: in systems not tying reimbursement to documentation comprehensiveness, AI scribing provides less marginal value. AI adoption is a symptom of a billing governance problem, not its solution.
5	“There should be AI‐generated notes to appease our billing overlords. And then a section where physicians can just free‐text pertinent information for other physicians.”	Commentator 54 T27: AI generated A&P are hot garbage Score: 5	“Billing overlords” delegitimise billing‐driven documentation by naming the institutional power relation producing it. Two‐tier note proposal acknowledges AI documentation may satisfy regulatory requirements while remaining insufficient for clinical communication.
6	“The ultimate problem is that, “saving the system money” means losing revenue. A full set of labs twice a month is ridiculously wasteful, but all that waste goes right into the hospital's pockets.”	Commentator 55 T1: Open Notes—Open Bills need to happen too Score: 102	Names the structural disincentive making genuine fiscal transparency threatening to institutional interests. Both OpenNotes and AI scribing operate within an economy that rewards documentation volume over quality—systematic pressure against integrity improvements.
7	“In Canada, we are mostly self‐employed. Cost is a big deal. An AI scribe programme is worth it. But is paying double for a “better” one worth the relative cost? It's like any other business decision.”	Commentator 56 T13: I tried a lot of AI medical scribes Score: 2	“It's like any other business decision” positions AI adoption outside the clinical governance framework, within practice management economics. Implies accountability and quality governance norms are perceived as business compliance requirements rather than professional obligations.
8	“I see 30 a day with an AI scribe—definitely makes me more productive and the notes are almost a little too good…”	Commentator 57 T107: Seeing 30 patients a day with AI scribes? Score: 5	“Almost a little too good” implies AI notes may over‐document, generating billing‐code‐satisfying prose for visits that did not warrant it. The governance implication—potential inadvertent upcoding—was approached but not explicitly named in any thread.
9	“They cut down on documentation time. That time is translated to more time with the patient. Overall, net zero saving time specifically. Patients like it.”	Commentator 58 T104: Do AI scribes help with documentation time? Score: 28	“Net zero saving time” is a precise economic characterisation: efficiency gains consumed by increased patient interaction. “Patients like it” introduces patient satisfaction as a fiscal governance consideration—experience scores have real revenue implications in value‐based care.
10	“She asked him to change his note from, “drinks 3 beers per day” to “drinks socially.” He respectfully declined and she was livid. Imagine a poor hospitalist involved in this mess.”	Commentator 59 T1: Open Notes—Open Bills need to happen too Score: 31	Documentation shapes insurance coverage, disability determinations, and care eligibility. When patients read their notes, they have an incentive to contest characterisations affecting their economic interests. AI pattern‐matching is particularly vulnerable to this challenge.
11	“OpenNotes does not increase the likelihood of a malpractice suit. Our insurer, CRICO, actively supports research on how OpenNotes can improve patient safety and has not changed coverage accordingly.”	Commentator 60 T2: AMA—OpenNotes clinicians and researchers Score: 5	Insurer actuarial endorsement as a fiscal governance authority. Notably, this endorsement has not been extended to AI‐generated OpenNotes—the fiscal risk profile of AI‐authored patient‐readable documentation has not been actuarially assessed.
12	“We've been open notes for 3 years or so. No sky has yet fallen. I haven't changed my documentation at all. In general, patients don't read my notes.”	Commentator 61 T1: Open Notes — Open Bills need to happen too Score: 15	“No sky has yet fallen,” closes the debate by pointing to the absence of catastrophic failure. Low patient engagement with notes reduces the patient‐side governance check on documentation quality — a governance gap that differs from, but matches the severity of, the feared avalanche of complaints.

Comparative comments from outside the United States sharpened this interpretation. One Canadian participant remarked that AI scribes “don't save me noticeable time personally” (Q4), explicitly linking this to a setting in which routine encounters do not require extensive documentation for reimbursement. Although such comments cannot establish causation, they suggest that the perceived usefulness of AI scribes is context‐dependent and may be amplified in systems where documentation is closely tied to payment. Participants also described AI‐generated text as serving institutional documentation demands, with one comment proposing separate AI‐generated notes to satisfy “billing overlords” alongside free text for physician‐to‐physician communication (Q5). This framing positions AI scribes not simply as neutral efficiency tools, but as instruments for navigating the administrative and economic logics of record production.

At the same time, clinicians expressed unease about the consequences of highly polished, expansive AI‐generated documentation. One participant described the notes as “almost a little too good” (Q8), raising concern that algorithmic over‐documentation may produce records that are more comprehensive, code‐ready, or administratively useful than the substance of the encounter would appear to warrant. The issue here is not that upcoding was directly demonstrated in the corpus, but that participants perceived a tension between documentation optimisation and faithful representation of clinical reality. This concern aligns with wider anxieties in the dataset about note inflation, standardisation, and the displacement of clinician judgement by administratively useful prose.

OpenNotes further complicates these dynamics by exposing economically consequential documentation to patient view. When patients can read their records, descriptions of behaviour, symptoms, or social history may be contested not only because they feel inaccurate or stigmatising, but because they may carry perceived implications for insurance, eligibility, or future care. The example of a patient disputing how alcohol use was recorded (Q10) illustrates how documentation can become a site of economic as well as relational tension. Taken together, the discourse points to a structural misalignment: AI scribes may help organisations and clinicians meet demanding documentation requirements, but the same expansive, billing‐oriented record can generate friction when made visible to patients. Clinicians are therefore positioned between institutional pressure for comprehensive, billable documentation and the communicative demands of patient‐facing transparency.

## 4 Discussion

This study shows that clinicians do not discuss AI‐generated clinical notes simply as a documentation convenience. They negotiate them as a governance problem at the intersection of accountability, epistemic reliability, patient visibility, privacy, and institutional incentive structures. In the OpenNotes era, this distinction matters because patients are no longer only reading what clinicians intentionally wrote; they are increasingly reading text that may have been inferred, condensed, or reformulated by ambient AI systems before full human verification. This extends the OpenNotes literature, which has generally associated note‐sharing with improved recall, engagement, medication management, and relational trust, while also documenting concerns about offence, confusion, and altered writing practices [1, 2, 4, 26, 27].

The strongest conceptual lens for interpreting these findings is contingent transparency. Atiku, Okoh, and Olakotan argue that transparency reforms are too often collapsed into a single category, producing what they call construct slippage. They distinguish Passive Transparency from reforms that add VOICE or TEETH, and show that disclosure alone rarely changes frontline outcomes unless it is connected to participation or credible enforcement. Read through that framework, OpenNotes in the age of AI scribing often looks like passive transparency. Patients can read the note, but they may lack timely and authoritative means to correct hallucinated details, challenge speaker misattribution, or trigger meaningful remediation once inaccurate AI‐generated text enters the legal record. That helps explain a central finding of this study: transparency does not itself resolve governance tensions but instead relocates them into the unstable gap between visibility and enforceability [17].

This framing clarifies Theme 1, the accountability vacuum. Across the corpus, clinicians repeatedly positioned the signature as the decisive liability transfer point. Yet they did not describe this as a robust safeguard. Instead, they narrated human review as mandatory in principle but structurally weakened in practice. That finding complicates a recurring assumption in the AI ethics literature: that human oversight can reliably function as the final safety mechanism for clinical AI [28, 29, 30]. In our dataset, oversight is undermined by time pressure, plausible‐sounding output, and limited traceability of error. Under OpenNotes, these problems intensify because fabricated commitments, altered speaker attribution, and incorrect treatment rationales become patient‐visible components of the medical record. The clinician's signature, therefore, appears less as evidence of meaningful control than as a legal shock absorber for institutional and technological uncertainty.

Theme 2, hallucination as governance crisis, further supports this interpretation. Clinicians in the corpus did not treat hallucination as a rare bug; they described it as a chronic epistemic condition of current AI documentation systems, especially in complex, specialist, or multi‐speaker encounters. Recent literature aligns with that concern. Studies and reviews on AI‐generated clinical documentation report mixed gains in efficiency and readability but recurring worries about factual inaccuracies, omissions, hallucinations, loss of nuance, and reduced performance in complex cases [31, 32, 33, 34, 35]. The AI‐scribe usability review by Atiku, Olakotan, and Owolanke is especially important here because it shows that AI scribes may reduce cognitive load and speed documentation while still being undermined by frequent errors, excessive note length, poor integration, and editing burdens that can erase time savings. It also reports that usability is better in routine or protocol‐driven encounters than in more complex workflows, closely matching the risk gradient described by clinicians in this study [13].

The EHR usability paper deepens this argument by showing that AI review is being layered onto an already fragmented documentation ecology rather than a neutral workflow. Olakotan et al. identify task‐switching, prolonged navigation, fragmented information, duplicated documentation, workarounds, and high cognitive load as major contributors to documentation burden. They also report poor EHR usability ratings, including a median System Usability Scale score of 45.9/100, with lower usability associated with greater burnout risk. That matters because the clinician expected to detect AI omissions, misattributions, or hallucinations is already working within a high‐friction documentation environment. In that setting, the governance demand to “just proofread it” becomes far less realistic than legal doctrine implies [36].

Theme 3, concerning note quality and clinical voice, shows that AI does not simply change who writes the note; it changes what the note is for. Prior OpenNotes and mental‐health literature suggest that patients often benefit from access through better understanding and engagement, but clinicians also worry about offence, confusion, stigma, and changes in therapeutic or documentation practice [37, 38, 39, 40]. Our findings suggest that ambient AI intensifies this dual‐audience problem. AI‐generated prose may satisfy institutional preferences for completeness, fluency or billing adequacy while weakening the note's communicative value for colleague‐readers and patient‐readers alike. This concern also aligns with broader work showing that stigmatising or invalidating language in records can transmit bias, erode trust, and harm marginalised patients [41, 42, 43]. AI‐generated notes may therefore standardise and scale representational harms that were already present in human‐authored records.

Theme 4, on privacy and data sovereignty, shows that clinicians in the corpus were not reassured by generic claims of compliance. They repeatedly distinguished formal legality from substantive stewardship, especially where retention policies, de‐identification, secondary use, and vendor access were concerned. That scepticism is consistent with wider health‐data ethics scholarship, which argues that older privacy and consent frameworks fit poorly with AI ecosystems shaped by opaque reuse and complex commercial arrangements [29, 44, 45]. Reviews of patient consent preferences similarly show that willingness to share health information depends on understandable explanations, trustworthy governance, and meaningful oversight [46, 47]. Our contribution is to show that clinicians themselves experience a governance vacuum at the point of care: they are expected to explain data flows and reassure patients even when institutional scripts and regulatory categories remain underdeveloped.

Finally, Theme 5 shows that AI documentation is embedded in a broader political economy of charting burden rather than standing outside it. Long before generative AI, physicians were already spending large amounts of time in the EHR and after hours, with longer notes associated with greater burden and lower efficiency [48, 49, 50]. The fiscal‐credibility review by Atiku and Olakotan sharpens this point by arguing that digital tools generate meaningful value only when apparent efficiencies can be translated into credible, governable economic outcomes. Applied here, that means AI notes should not be judged only by whether they feel faster. The more difficult question is whether they produce economically legible value without simultaneously inflating documentation, shifting hidden verification work onto clinicians, or weakening the credibility of the record as an account of what actually happened in the encounter [19].

In conclusion, the clinician discourse analysed here suggests that AI‐generated notes in the OpenNotes era are governed through a fragile fiction: that disclosure plus signature equals accountability. The contingent‐transparency literature shows why that fiction persists; the usability literature shows why it fails in practice; and the fiscal‐credibility literature shows why institutions may still find adoption attractive despite unresolved risks. The central governance challenge is therefore not simply how to make AI notes faster, but how to redesign transparency, accountability, workflow conditions, and economic incentives so that patient‐facing records remain genuinely trustworthy.

## 5 Conclusion

This study shows that the integration of ambient AI scribes into the OpenNotes environment creates a significant governance problem. Clinicians do not describe algorithmic documentation as a straightforward administrative convenience. Instead, they frame it as a structural risk arising from tensions between accountability, epistemic reliability, patient visibility, privacy, and institutional incentives. Driving this problem is a continuing regulatory reliance on passive transparency. Current institutional practice often assumes that patient access to notes, combined with a clinician's digital signature, is sufficient to secure accountability. The discursive evidence examined here challenges that assumption directly.

What emerges instead is an interrelated set of systemic vulnerabilities. When algorithmic tools generate plausible hallucinations, obscure the processes through which information is extracted and reformulated, and expand clinical text in ways that serve billing or organisational demands, the medical record begins to lose both epistemic reliability and fiscal credibility. Expecting already overburdened clinicians to detect these subtle distortions consistently is unrealistic. Under such conditions, the professional signature functions less as evidence of meaningful oversight than as a legal safeguard against institutional and technological uncertainty. Patient access, on its own, does not resolve this problem. Where no authoritative route for correction exists, visibility offers only a limited and potentially misleading form of empowerment. The tensions remain. They are relocated into the gap between what patients can read and what they are actually able to challenge.

This suggests a fundamental shift in how healthcare systems must approach documentation governance. Policymakers and institutional leaders can no longer rely on the fiction that human‐in‐the‐loop review provides sufficient oversight for complex and opaque forms of algorithmic generation. Future frameworks need to move beyond passive disclosure towards stronger models of contingent transparency. That shift requires explicit VOICE mechanisms through which patients and clinicians can contest inaccuracies, misattributions, and disputed content using clear and responsive institutional pathways. It also requires TEETH in the form of provenance tracking, auditability, enforceable vendor accountability, and robust standards for data stewardship, clinical safety, and review conditions. Until such participatory and enforcement structures are established, AI‐mediated documentation is likely to continue advancing institutional efficiency and revenue goals without adequately protecting clinical truth, record integrity, or patient trust.

## Funding

The authors have nothing to report.

## Ethics Statement

Ethical approval for this study was granted by Aston University.

## Conflicts of Interest

The authors declare no conflicts of interest.

## Data Availability

The data generated and analysed during the current study are not publicly available. The study drew on publicly accessible Reddit material, but the full dataset is not shared because of the ethical and privacy considerations associated with potentially identifiable online content. Quoted material has therefore been used selectively and with caution to reduce traceability.
